# No Tangible Effects of Field-Grown Cisgenic Potatoes on Soil Microbial Communities

**DOI:** 10.3389/fbioe.2020.603145

**Published:** 2020-11-03

**Authors:** Sascha M. B. Krause, Astrid Näther, Vilma Ortiz Cortes, Ewen Mullins, Geert J. T. Kessel, Lambertus A. P. Lotz, Christoph C. Tebbe

**Affiliations:** ^1^Thünen Institute of Biodiversity, Federal Research Institute for Rural Areas, Forestry and Fisheries, Braunschweig, Germany; ^2^Zhejiang Tiantong Forest Ecosystem National Observation and Research Station, Center for Global Change and Ecological Forecasting, School of Ecological and Environmental Sciences, East China Normal University, Shanghai, China; ^3^Teagasc Crops, Environmental and Land Use Program, Crop Science Department, Oak Park Crops Research Centre, Carlow, Ireland; ^4^Plant Research International, Wageningen University & Research, Wageningen, Netherlands

**Keywords:** genetically modified plants, field trial, environmental risk assessment, nitrite reductase genes (*nirS*) (nirK), 16S rRNA gene, fungal ITS sequence, soil DNA analyses, potato rhizosphere

## Abstract

DNA modification techniques are increasingly applied to improve the agronomic performance of crops worldwide. Before cultivation and marketing, the environmental risks of such modified varieties must be assessed. This includes an understanding of their effects on soil microorganisms and associated ecosystem services. This study analyzed the impact of a cisgenic modification of the potato variety Desirée to enhance resistance against the late blight-causing fungus *Phytophthora infestans* (Oomycetes) on the abundance and diversity of rhizosphere inhabiting microbial communities. Two experimental field sites in Ireland and the Netherlands were selected, and for 2 subsequent years, the cisgenic version of Desirée was compared in the presence and absence of fungicides to its non-engineered late blight-sensitive counterpart and a conventionally bred late blight-resistant variety. At the flowering stage, total DNA was extracted from the potato rhizosphere and subjected to PCR for quantifying and sequencing bacterial 16S rRNA genes, fungal internal transcribed spacer (ITS) sequences, and *nir* genes encoding for bacterial nitrite reductases. Both bacterial and fungal communities responded to field conditions, potato varieties, year of cultivation, and bacteria sporadically also to fungicide treatments. At the Dutch site, without annual replication, fungicides stimulated *nirK* abundance for all potatoes, but with significance only for cisgenic Desirée. In all other cases, neither the abundance nor the diversity of any microbial marker differed between both Desirée versions. Overall, the study demonstrates environmental variation but also similar patterns of soil microbial diversity in potato rhizospheres and indicates that the cisgenic modification had no tangible impact on soil microbial communities.

## Introduction

Potato is the world’s most important non-cereal food crop, and *Solanum tuberosum* L. by far is its most widely cultivated species in modern agriculture (Birch et al., 2012). Its productive cultivation, however, is increasingly threatened by nematodes and microbial pathogens, the latter including viruses, bacteria, protists, and fungi. In traditional potato growing regions as they exist in Northern Europe, the oomycete *Phytophthora infestans*, causing late blight, is in this regard of paramount importance. To control this devastating disease, mixtures of chemical fungicides are applied during their cultivation, often as regularly as every 4 days (Haverkort et al., 2009; Cooke et al., 2011). Such control measures are not only an economical concern, costing millions of Euros each year, but also an ecological hazard, and thus, in the longer term, not sustainable: Fungicides applied on potato fields may affect other non-target soil microorganisms and they may also not be completely degraded by microbial processes during a growing cycle, thereby causing the risk of their accumulation and translocation to other fields or ecosystems (Niemi et al., 2009; Stenrod, 2015; Hakala et al., 2020).

For limiting both, the economic loss and environmentally adverse effects of fungicides, the development of new *Phytophthora*-resistant potato varieties is of high priority (Park et al., 2009). Wild potato species have played an important role in potato breeding for resistance (Vleeshouwers et al., 2011), but the development of new elite varieties with resistance genes (*R* genes) by classical breeding programs is slow and may take decades (Barrell et al., 2013). Molecular genetic modification techniques, on the other hand, allow for the stacking of multiple *R* genes into already established varieties, thereby enhancing chances to counteract the speed and efficacy of evolutionary adaptations of the fungal pathogen. This can give rise to more durable varieties that can be cultivated with a much lower fungicide demand (Haverkort et al., 2016; Lotz et al., 2020).

In the European Union (EU), new varieties generated by molecular methods fall under the definition of genetically modified organisms (GMOs) according to Directive 2001/18/EC, even if they exclusively contain DNA from other genetically compatible plant species, as the case with potato and its relatives. Such constructs are not transgenic but cisgenic (Haverkort et al., 2008). Regulation in modern agriculture, whether in Europe or elsewhere, requires that risks associated with the release of genetically modified (GM) plants into the environment must be assessed, encompassing potential adverse effects on humans, animals, and environment (Sanvido et al., 2007). The environmental assessment addresses several areas of risks, including interactions with non-target organisms and effects on biogeochemical processes, both of them considering soil as the main receiving environment (EFSA Panel on Genetically Modified Organisms, 2010). A special focus of such analyses relates to the rhizosphere, i.e., the soil affected by roots during cultivation of the plants, as this represents a spatial soil compartment with the highest probability of exposing products of the GM plant to soil organisms.

Consequently, numerous experimental field studies have been conducted during the last decades analyzing the response of microbial communities inhabiting the rhizosphere of many different genetically modified crops (Liu et al., 2005; Guan et al., 2016). To include all microbial community members, independent of their culturability in laboratory growth media, such studies were typically done by characterizing the structural diversity of the communities based on PCR-amplified prokaryotic 16S rRNA, eukaryotic18S rRNA genes, or fungal chromosomal internal transcribed spacer (ITS) regions from soil-extracted DNA. While some studies detected certain shifts in the microbial community structure linked to the genetic modification, the majority of them did not (see, e.g., reviews Bruinsma et al., 2003; Liu et al., 2005; Guan et al., 2016). In fact, from most studies in which GM and conventionally bred varieties were compared to each other, the authors concluded that the variations in microbial community compositions caused by field sites, seasons, or plant age were greater than those triggered by the genetic modifications (Heuer et al., 2002; Baumgarte and Tebbe, 2005; Hannula et al., 2012). However, great differences exist between the different studies about the sensitivity of their detection methods and intensity of monitoring, e.g., regarding replication within and between field sites, the frequencies of sampling, and annual repetitions. Concerning the monitoring tools, early studies utilized genetic fingerprinting techniques (Smalla et al., 2007; Liu et al., 2008; O’Callaghan et al., 2008) and focused on sequencing selected dominant community members (Heuer et al., 2002; Schmalenberger and Tebbe, 2002; Milling et al., 2004), while more recently, the potential of high-throughput sequencing technologies for PCR amplicon analyses was applied to also search for differences between less dominant microbial community members. However, for the cases analyzed so far, including GM maize and cotton, such more sensitive methods did not reveal new hazards (Dohrmann et al., 2013; Qi et al., 2018; Szoboszlay et al., 2019). High-throughput amplicon sequencing was also recently applied to study potatoes, and together they suggest the existence of a plant-specific core rhizomicrobiome, which encompasses a stable and a dynamic substructure (Pfeiffer et al., 2017; Mardanova et al., 2019; Hou et al., 2020). However, there is yet no information on how transgenic or cisgenic modification may affect these communities.

The objective of this study was therefore to analyze the effect of a cisgenic modification conferring late blight resistance by the insertion of *R* genes from *Solanum venturii* to the susceptible widely cultivated potato variety Desirée on rhizosphere-inhabiting soil microbial communities (fungi, bacteria). The modified potatoes were cultivated with annual replication at two field sites in typical Atlantic European growing regions, one located in Ireland and one in the Netherlands, in experimental fields along with the non-modified variety and the conventionally bred late blight-resistant variety Sarpo Mira (SM). Treatments without and with the application of fungicides to control *P. infestans* were included. Bacterial and fungal communities were analyzed for their abundance and structural diversities. To detect a potential impact on biogeochemical processes, i.e., the nitrogen transformation potentials in the rhizospheres, bacterial *nirS* and *nirK* genes encoding for two alternative versions of the enzyme nitrite reductase were also analyzed.

## Materials and Methods

### Plant Varieties of This Study

Three potato varieties (*Solanum tuberosum L.*) were investigated in this study: (1) SM, a conventionally bred variety with high resistance against the late blight pathogen *P. infestans*; (2) Desirée, a conventionally bred widely cultivated variety highly susceptible to *P. infestans*; and (3) Desirée clone A15-031, a genetically modified derivative of Desirée with resistance to the late blight through chromosomal transformation of the *Rpi-vnt1.1* gene from *S. venturii* (Foster et al., 2009; Haverkort et al., 2016). The gene is only expressed at exposure to *P. infestans*. Considering that *S. tuberosum* and *S. venturii* are crossable species, the modified A15-031 is considered to be cisgenic. Their use in Europe falls under the EU Directive 2001/18 (Jacobsen and Schouten, 2008).

### Field Sites and Experimental Design

Soil samples originated from two field trials set up for the EU-funded AMIGA project (Assessing and Monitoring the Impacts of Genetically modified plants on Agro-ecosystems). The field trials were performed with the permission of the respective national governments (License no. B/IE/12/01 and Permit IM10-006, respectively).

One field site (OP) was located in Ireland (Oak Park, Carlow; 52°51′21.8′′N and 6°54′43.6′′W), the other (VM) in the Netherlands (Valthermond; 52°52′18.6′′N and 6°56′33.6′′E) in the years 2013 and 2014. Soil properties at both sites and the experimental field designs have been described elsewhere (Lazebnik et al., 2017; Kessel et al., 2018). In brief, the soil of OP was boulder clay, while the soil of VM predominantly consisted of reclaimed peat soil (90.1% sand, 9.9% organic matter). The soils of each field plot at the time of sampling in OR and VM were analyzed for their pH, total soil organic carbon (C), and total nitrogen (N_*tot*_), as previously described (Poeplau et al., 2016).

The experimental field sites consisted of field plots in randomized block design. The plot sizes at OP were 3 m × 3 m, while they were 6 m × 6 m in VM. At both sites, the plots were separated by interspaces of 6-m grass on each side. All three potato varieties were seeded and cultivated in 2013 and 2014 according to agricultural practice. More details on the agricultural procedures can be found elsewhere (Kessel et al., 2018). Planting was completed in OP on June 4, 2013, and May 18, 2014, and in VM on June 10, 2013, and May 21, 2014, respectively. The whole field experimental design included a total of three different treatments in respect to control of late blight: unsprayed (no fungicides), sprayed at a weekly schedule (fungicides; common practice), and an integrated pest management strategy (IPM 2.0) (see Kessel et al., 2018). The IPM 2.0 however was not considered in this study. At the field site in OP, each variety and treatment were replicated on 12 plots and in VM on seven plots. The fungicides applied on both sites were the same and included the active compounds cymoxanil, propamocarb, cyazofamid, and fluopicolide, respectively (Kessel et al., 2018).

### Soil Sampling

Rhizosphere samples from the field-grown potatoes were collected at BBCH 65, i.e., during their flowering period, which was between July and August for both years. In total, 42 plots were sampled at each site for each year: seven replicated plots for each potato variety with or without application of fungicides were sampled. The field site VM, however, was in secret destroyed in 2013 by anonymous persons without any claims, thereby strongly reducing the number of plots and samples that could be analyzed. For this study and sound statistical analyses, we excluded the data from VM for 2013.

To collect the rhizosphere, three individual plants were carefully dug out during afternoon with a digging fork from each plot, placed into plastic bags, and transferred within 4 h in a temperature-controlled box (ca. 12°C) to the laboratory. Here, loosely adhering soil was removed from the roots by manually shaking for ca. 1 min. The soil adhering to roots was defined as rhizosphere, and it was detached for each sample by suspending 8 g of fresh root material in 30 ml of sterile saline for 30 min at 4°C in an orbital shaker (Model 3040, GFL, Burgwedel, Germany) at 10 rpm followed by centrifugation at 4,100 × *g*. The resulting pellets that included the microbial cells with their DNA were stored at −80°C for subsequent analyses.

### DNA Extraction

DNA was extracted from frozen cell pellets using the FastDNA SPIN kit for soil (MP Biomedicals, Illkirch, France) with the following modifications: The extraction included two bead-beating steps (45 s at 6.5 m s^–1^) on a FastPrep-24 instrument (MP Biomedicals, Eschwege, Germany) and additional washing of the binding matrix with 1 ml 5.5 M guanidine thiocyanate (Carl Roth, Karlsruhe, Germany) until the matrix regained its original color. DNA was quantified by using a Thermo Scientific NanoDrop Spectrophotometer (Model 2000c, Peqlab, Erlangen, Germany). Extracted DNA was stored at −20°C for subsequent analyses.

### Quantitative PCR

Bacterial and archaeal 16S rRNA genes, nitrite reductase genes (*nirK* and *nirS*), and fungal ITS regions were quantified in duplicates from soil DNA using the StepOnePlus Real-Time PCR System (Life Technologies GmbH, Darmstadt, Germany). Detailed sequence information of primers and probes is listed in Supplementary Table S1. The soil DNA was diluted 10- and 50-fold, and subsequently, 2 μl of these diluted DNA extracts were used in 20-μl real-time PCR reaction volume.

Bacterial and archaeal 16S rRNA genes were quantified by using the Maxima Probe qPCR ROX Master Mix (Thermo Fisher Scientific, Epsom, United Kingdom) with 0.5 μM primers and 0.2 μM FAM-labeled *Taq*Man probes. The temperature profile was 10 min of denaturation at 95°C followed by 40 cycles of 95°C for 15 s and 60°C for 60 s.

Both *nirK* and *nirS* genes as well as fungal ITS regions were quantified by using the Maxima SYBRGreen/ROX qPCR Master Mix (Thermo Fisher Scientific). In the case of *nirK* and *nirS* genes, the reaction was supplemented with additional MgCl_2_ to reach a final concentration of 1 mM. The temperature profile was 10 min of denaturation at 95°C followed by 40 cycles of 95°C for 15 s, 52°C for 30 s, and 72°C for 60 s for fungal ITS, and 10 min of denaturation at 95°C followed by 40 cycles of 95°C for 15 s, 63°C for 30 s decreased by 1°C in each cycle to 58°C, and 72°C for 60 s for *nirK* and *nirS*.

All reactions were validated by melt curve analyses. Standard curves were prepared from 10-fold dilutions of clones generated with the pGEM^®^ -T Vector System (Promega, Mannheim, Germany). The following reference organisms were used: *Bacillus subtilis* (16S rRNA gene, Bacteria), *Methanobacterium oryza* (16S rRNA gene, Archaea), *Fusarium culmorum* (ITS region, Fungi), *Pseudomonas stutzeri* (*nirS* gene, Bacteria), and *Sinorhizobium meliloti* (*nirK* gene, Bacteria).

The amplification efficiencies were 95.3% ± 3.2% for bacterial 16S rRNA genes, 97.4% ± 0.5% for archaeal 16S rRNA genes, 85.8% ± 1.2% for fungal ITS regions, 87.5% ± 2.9% for *nirK* genes, and 92.6% ± 2.1% for *nirS* genes. Results of qPCR were log10 transformed and normalized per ng extracted DNA.

### Illumina MiSeq Sequencing and Sequence Analysis

The V4 region of bacterial and archaeal 16S rRNA genes and a fragment of the *nirK* gene were sequenced by Illumina MiSeq dual-index sequencing strategy as previously described (Kozich et al., 2013). Detailed gene-specific primers that were used to target 16S rRNA genes and *nirK* gene fragments, respectively, are listed in Supplementary Table S1. PCR amplifications were performed from each sample by using the FastStart High Fidelity PCR System (Roche Diagnostics, Mannheim, Germany) in 50-μl reaction volume including the following templates and reagents: 1 μl DNA-extract, 0.4 μM of each primer, 200 μM of each dNTP, 5% dimethyl sulfoxide, 1.8 mM MgCl_2_, and 2.5 U FastStart High Fidelity Enzyme Blend. The temperature profile started with initial denaturation at 95°C for 2 min followed by 35 cycles of 95°C for 30 s, 50°C for 30 s, and 72°C for 60 s and finishing with 72°C for 5 min. Two PCRs were performed for each target gene, pooled, purified with HiYield PCR Clean-up & Gel-Extraction kit (SLG), and subsequently quantified with Quant-iT PicoGreen dsDNA assay (Invitrogen, Darmstadt, Germany). Equimolar amounts of the PCR products were then pooled for each target and sent to StarSEQ (Mainz, Germany) for sequencing. Both the 16S rRNA gene and *nirK* PCR products were sequenced each in a single MiSeq run with 600 cycle V3 chemistry (300-bp paired-end).

ITS 1 and ITS 2 regions of fungi were sequenced by using specific primers with Illumina overhang adaptors (Supplementary Table S1). PCR amplifications were carried out from each sample by using the Kapa2G Robust PCR ReadyMix (Kapa Biosystems, Wilmington, MA, United States) in 50-μl reaction volumes including the following templates and reagents: 10 ng DNA extract and 0.2 μM of each primer. The temperature profile started with an initial denaturation at 95°C for 3 min followed by 35 cycles of 94°C for 45 s, 62°C for 45 s, and 72°C for 60 s and finished with 72°C for 7 min. Agencourt AMPure XP beads (Beckman Coulter, Brea, CA, United States) were used to clean up the PCR products. In an additional limited-cycle PCR, sequencing adapters and index sequences were added: 5 μl purified PCR product and primers from the Nextera XT Index kit (Illumina). Cycling conditions: initial denaturation at 95°C for 3 min followed by eight cycles of 95°C for 30 s, 55°C for 30 s, and 72°C for 30 s and finally 72°C for 5 min. After purification, as described above, equimolar amounts of PCR products were sent to the DNA Sequencing Facility of Teagasc (Oak Park, Carlow, Ireland) for sequencing in two MiSeq runs with 600 cycle V3 chemistry. In the first run, samples from Ireland 2013 and, in the second run, samples from Ireland and the Netherlands in 2014 were sequenced. All sequences are available at the European Nucleotide Archive (ENA) under accession number PRJEB39718.

The DADA2 pipeline version 1.8.0 in R 3.5.2 was used to process raw sequence as described in detail elsewhere (Callahan et al., 2016; R Core Team, 2018). In this study, different target regions were processed separately with the following modifications:

The 16S rRNA gene forward and reverse reads were trimmed at positions 280 and 120 from the end or at any position with a *Q*-score of 2 or lower. Forward reads over two expected errors and reverse reads over five expected errors or any ambiguous bases were discarded. Error models were constructed with a total number of 2e + 08 bases and random sample picking. Resulting sequence variances (SVs) were taxonomically classified by using the SILVA reference version 128 (Pruesse et al., 2007). SVs that were longer than 280 nucleotides, or identified as mitochondrial or chloroplast sequences, or not classified into *Bacteria* or *Archaea* were removed from the dataset.

The *nirK* gene forward and reverse reads were trimmed at position 10 from the start and positions 290 and 250 from the end or at any position with a *Q*-score of 2 or lower. Forward reads over two expected errors and reverse reads over five expected errors or any ambiguous bases were discarded. Error models were constructed with a total number of 1e + 08 bases and random sample picking. In the sample interference algorithm, the band size parameter of the DADA2 algorithm was set to 32. SVs were translated into amino acid sequences using FrameBot (Wang et al., 2013) to account for frameshifts. Resulting translated sequence variants (TSVs) were compared to a *nirK* reference database at the FunGene repository (Fish et al., 2013) with a minimal length cutoff of 80 amino acid residues and identity cutoff 0.8.

The DADA2 ITS pipeline version 1.8.0 in R 3.5.2 was used to process raw ITS sequences (Pauvert et al., 2019). First primers were removed from fungal ITS forward and reverse reads by using cutadapt version 1.18 (Martin, 2011). Forward and reverse reads over two expected errors and those shorter than 50 nucleotides were discarded. Reads were truncated at the first position with a *Q*-score of 10 or lower. In the sample interference algorithm, the band size parameter of the dada algorithm was set to 32. Run variation can influence the error model. Therefore, each sequencing run was separately processed and merged into one sequencing table before chimera removal and taxonomic assignment. Resulting SVs were taxonomically classified by using the UNITE ITS database version 7.2 (Koljalg et al., 2013). All sequences that were identified as non-fungal were removed from the dataset.

### Statistical Analyses

R 3.5.2 (R Core Team, 2018) was used for all statistical analyses. The abundances of Bacteria, Archaea, and Fungi between potato varieties, sites, years of cultivation, and fungicide treatments within each year were compared with ANOVA and Tukey’s honestly significant difference (HSD) *post hoc* tests implemented in the R package stats. Simpson’s diversity index was calculated with R package ‘vegan’ version 2.5-2 (Oksanen, 2015). Significant differences were calculated using Kruskal–Wallis rank sum tests followed by pairwise Wilcoxon rank-sum tests with Benjamini–Hochberg correction for multiple testing and a false discovery rate (FDR) of 5% as implemented in the R package stats. Before these analyses, singletons and doubletons were removed from sequencing data sets.

Bacterial, archaeal, and fungal communities based on Illumina MiSeq sequencing were further analyzed using tools for compositional data analysis (Gloor et al., 2016, 2017). First, Bayesian-multiplicative replacement of count zeros (cmultRepl) was performed implemented in the R package zCompositions (Palarea-Albaladejo and Martín-Fernández, 2015) to treat zeros in compositional count data. Since high sparsity influences the performance of cmultRepl, 16S rRNA gene SVs, or *nirK* SVs, or fungal ITS SVs were removed that had less than 0.05, 0.10, or 0.10% relative abundance, respectively. Then, the centered log-ratio (CLR) transformation was applied, implemented in the R package compositions to correct for differences in sequencing depth between samples (Gloor et al., 2016). Differences in community composition were visualized by using non-metric multidimensional scaling (NMDS) based on Aitchison distance implemented in the R package vegan.

Vector fitting was used to depict the influence of environmental variables on different ordinations implemented in the R package vegan. Variation partitioning was used with redundancy analysis (RDA) to identify the variation explained by different explanatory variables implemented in the R package vegan. Significance was tested by using the ANOVA function in R on the RDA object with Monte Carlo permutations. The R package ggplot2 was used for more advanced graphics (Wickham, 2016). Variation in community structure in response to variety and fungicide treatment was assessed by using permutational multivariate analyses of variance (PERMANOVA) implemented in the R package vegan.

The R package ALDEx2 (Fernandes et al., 2014) was used to identify differentially abundant SVs and TSVs between potato varieties and fungicide treatments within each year. ALDEx2 was applied with Welch’s t test and correction for multiple testing with an FDR of 5%. Only *nirK* TSVs and fungal ITS SVs that had at least 0.10% relative abundance, or 16S rRNA gene SVs with at least 0.05% relative abundance, in any of the compared samples were included.

## Results

### Chemical Properties of the Rhizosphere Soils

Analyses of the potato rhizosphere soils at the time of sampling (growth stage BBCH 63, flowering) revealed that the pH at the Irish site OP was near neutral (6.5–7.1), while it was acidic at the Dutch site VM, within a range from 4.5 to 4.8 (see Supplementary Table S2). Soil organic C was higher in VM compared to OP, while soil nitrogen levels were similar, thus resulting in higher CN ratios at VM with ca. 24 compared to OP with ca. 12. Neither fungicide applications nor the cisgenic modification or the potato variety had a significant effect on the chemical properties of this root-affected soil compartment.

### Bacterial, Archaeal, and Fungal Abundance

**FIGURE 1:**
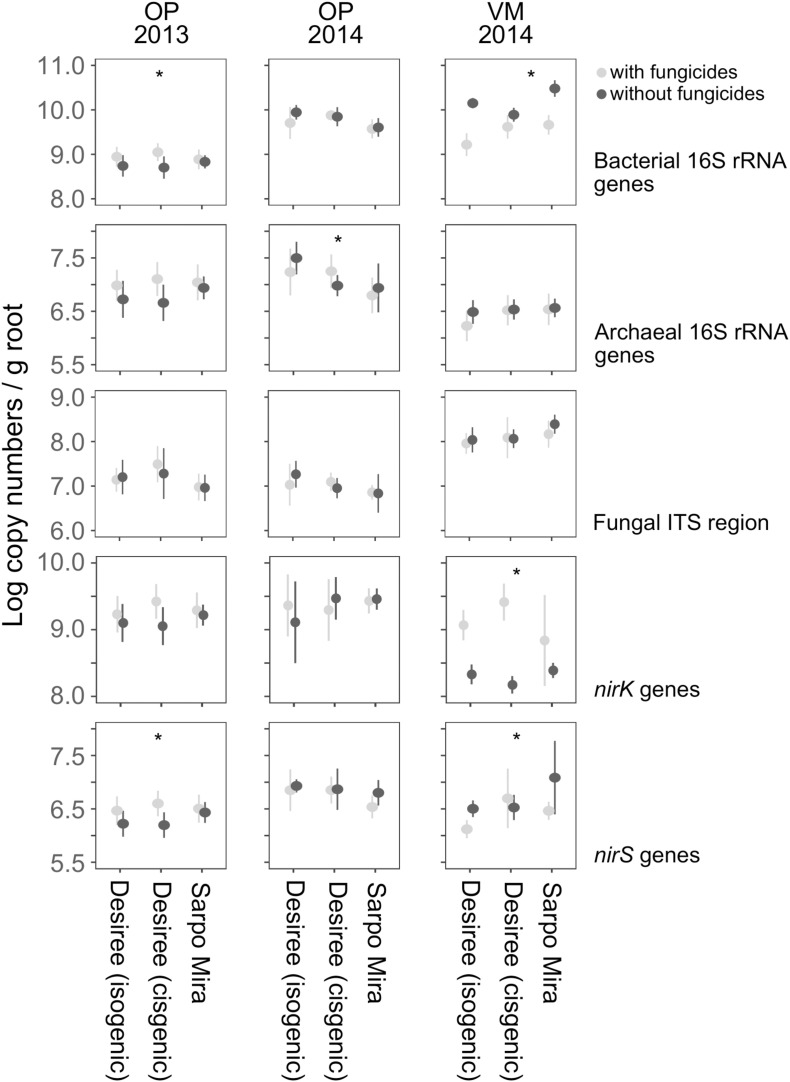
Log_10_ mean abundances of bacterial and archaeal 16S rRNA gene copies, fungal internal transcribed spacer (ITS) region copies, *nirK* and *nirS* gene copies in soil samples. Error bars indicate standard deviation (*n* = 7). Asterisks indicate significant differences between different cultivars and treatments for each target gene (*p < 0.05) based on ANOVA.

Independent of the sites (OP, VM) and sampling years (2013, 2014), the highest gene numbers detected from soil DNA were for *Bacteria* targeted by the 16S rRNA and *nirK* genes, respectively (Figure 1). The relative contribution of *Fungi* to the soil microbiome was higher at the Dutch site (VM) than at the Irish site (OP), while the proportion of *Archaea* appeared to be less. At both sites, the prokaryotic nitrite reductase *nirK* genes were one to two orders of magnitude more abundant than *nirS*. Several significant differences were detected between years, but differences between the potato varieties or fungicide treatments only occurred sporadically. The isogenic and cisgenic version of Desirée showed no differences, except for the fungicide-treated samples at the VM site in 2014, where it was significantly more abundant in response to the fungicides (*p* = 0.011). The same trend was seen for the non-modified Desirée and, though less pronounced, for SM, but both effects were not statistically supported. At OP in 2013, the abundance of bacterial 16S rRNA genes in rhizospheres of fungicide-treated plots of the cisgenic Desirée was higher compared to those not exposed to fungicides (*p* = 0.054). The same finding was observed for the *nirS* gene abundance (*p* = 0.038). This effect was not seen in 2014. Only in 2014, the archaeal 16S rRNA gene abundance was significantly higher in fungicide-untreated samples between the isogenic Desirée and the variety SM (*p* = 0.054). The cisgenic Desirée was intermediate between both.

In VM, the abundance of bacterial 16S rRNA genes in rhizospheres of fungicide-treated plots of the isogenic Desirée (*p* < 0.001) and SM (*p* < 0.001) was lower compared to those not exposed to fungicides, thus showing the opposite response to fungicides recorded at the Irish OP site the year before. Interestingly, at the VM site, the abundance of *nirK* showed the opposite effect, being higher in response to fungicides treatments, as seen with samples of the isogenic (*p* = 0.002) and cisgenic (*p* < 0.001) Desirée. Fungicide treatments resulted in significantly higher bacterial 16S rRNA gene abundance in cisgenic Desirée (*p* = 0.011) and SM (*p* = 0.003) compared to the isogenic Desirée. Similarly, untreated samples depicted significantly lower bacterial 16S rRNA gene abundances between SM and both the isogenic (*p* = 0.054) and cisgenic (*p* < 0.001) Desirée. For *nirS* gene abundance, treated samples were significantly lower with the cisgenic Desirée than untreated samples of SM (*p* < 0.001).

For fungal abundance in the potato rhizosphere, as indicated by their ITS genomic regions, neither the treatments nor the varieties or cisgenic modification or the annual replication had any tangible effect.

### Sequencing Yields and Phylogenetic Diversity of Prokaryotes and Fungi Detected in the Rhizosphere Samples

**FIGURE 2:**
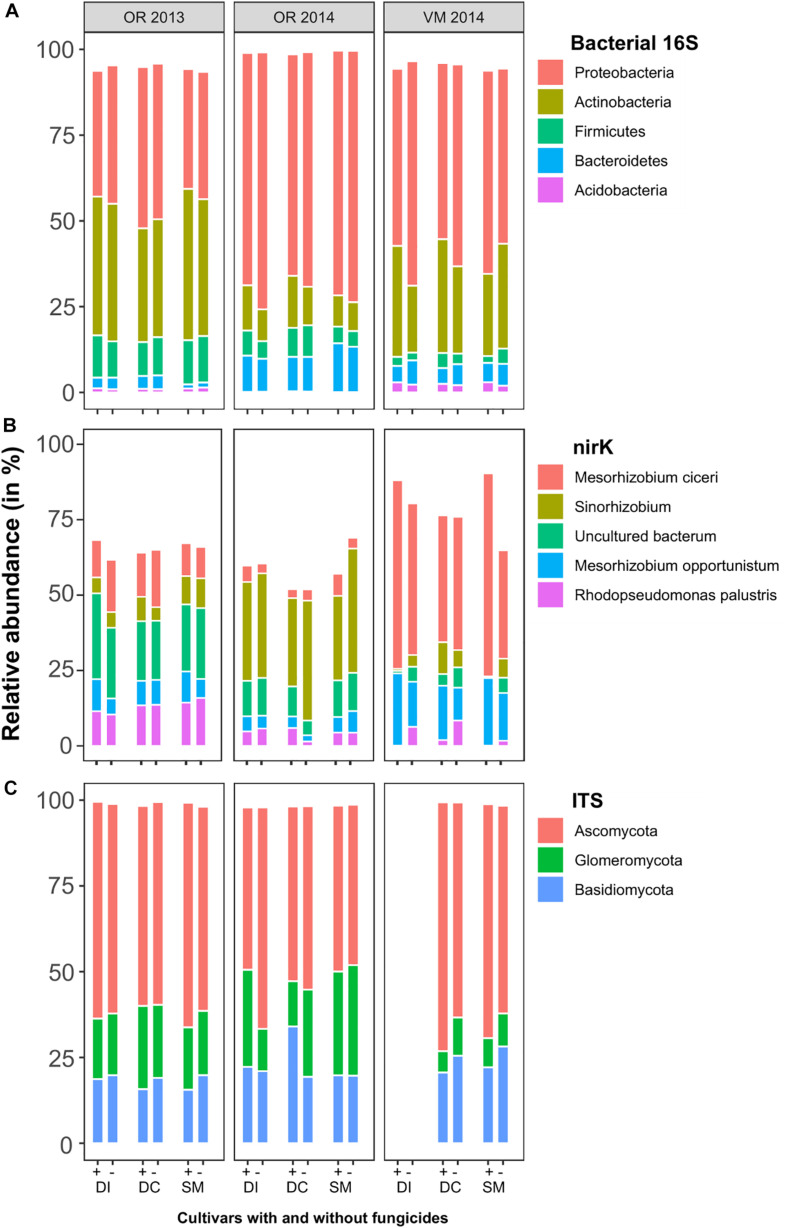
Relative abundances of dominant phyla (bacterial 16S rRNA, **(A)**; *nirK*, **(B)**) or genera [fungal internal transcribed spacer (ITS), **(C)**] in soil samples with and without fungicides (±) for the cultivars Desirée, both isogenic (DI) and cisgenic (DC), as well as Sarpo Mira (SM) at Oak Ridge (OR), Ireland, and Valthermond (VM), the Netherlands, in 2013 and 2014.

The number of high-quality bacterial and archaeal 16S rRNA sequences obtained from 126 samples (libraries) was 3,515,265, which grouped into 4,373 SVs. A sample included on average 27,899 ± 8,206 sequences, with the smallest library of 11,509 and the largest with 50,042 sequences. SVs were classified into 26 bacterial phyla and one archaeal phylum. A total of 99.7% of sequences were assigned to the domain Bacteria. The most abundant bacterial phyla were Proteobacteria (55.5% ± 15.2%), Actinobacteria (25.7% ± 13.9%), Firmicutes (7.1% ± 4.5%), Bacteroidetes (6.6% ± 4.9%), and Acidobacteria (1.2% ± 1.2%). Within the phylum Proteobacteria, the following abundance structure was identified: Gammaproteobacteria (23.2% ± 18.0%), Alphaproteobacteria (21.4% ± 5.9%), Betaproteobacteria (9.6% ± 6.4%), and Deltaproteobacteria (1.16% ± 1.0%). At the site in OP, the relative dominance of Proteobacteria was less pronounced in 2013 than in 2014 (Student’s *t*-test, *p* < 0.001) and instead the relative abundance of Actinobacteria and Firmicutes was higher (Student’s *t*-test, *p* < 0.001 and *p* < 0.001, respectively), thus indicating annual variation (Figure 2). This also applied to Bacteroidetes, which were more abundant in OP 2014 than in 2013 (Student’s *t*-test, *p* < 0.001). For site variation, Acidobacteria were more abundant in VM than in OP from both years (Student’s *t*-test, *p* < 0.001). There was no clear effect of applying fungicides or potato varieties on the relative abundance of the bacterial phyla at the 16S rRNA gene level.

Because of their quantitative dominance, the denitrifying community was characterized by sequencing the *nirK* but not the *nirS* gene amplicons. In total, 121 samples yielded in total 1,164,234 *nirK* amplicon sequences, which grouped into 4,147 SVs. Three samples of variety SM with fungicides, one sample of SM without fungicides, and one sample of variety Desirée cisgen (DC) without fungicides from VM (Netherlands) in 2014 had to be removed because of very low sequencing yields due to technical problems. FrameBot could match 2,907 SVs, containing 963,413 sequences, to a reference sequence in the FunGene *nirK* database (Fish et al., 2013). These SVs translated into protein sequences with an average amino acid residue length of 138 and matched with 39 reference sequences.

The most abundant *nirK*-bearing taxa were assigned to *Mesorhizobium ciceri* bv. *biserrulae* WSM1271 (22.1% ± 21.5%), *Sinorhizobium* sp. NP1 (15.3% ± 14.4%), *Mesorhizobium opportunistum WSM2075* (10.1% ± 6.4%), *Rhodopseudomonas palustris* (6.9% ± 5.1%), all being members of the Alphaproteobacteria (Figure 2). The relative abundance of *Sinorhizobium* sp. was much higher in OP soil in 2014 than in 2013, and an opposite trend was seen for *nirK* from *Rhodopseudomonas palustris*. A strong dominance of *M. ciceri* was seen in the Dutch VM soil, while this taxon was only a minor contributor at OP. Neither the potato varieties nor the cisgenic modification of even the application of fungicides had a notable effect at this level of phylogenetic resolution.

Sequencing of 106 samples for fungal ITS regions yielded 6,031,139 high-quality sequences, which grouped into 2,433 SVs (see Supplementary File 1). From the site VM, only samples of SM and the cisgenic Desirée were analyzed (DNA of the isogenic Desirée was lost during the sequencing run). Individual samples included on average 56,897 ± 27,144 sequences, with the smallest ITS library of 16,557 and the largest with 128,348 sequences. Taxonomically, the SVs could be assigned to 13 fungal phyla. The most abundant were Ascomycota (58.9% ± 7.6%), Basidiomycota (21.3% ± 4.6%), and Glomeromycota (18.5% ± 8.0%). A total of five SVs were classified as *Rhizaria* (eukaryotic protists) and one SV could not be assigned at all. These six SVs were removed from the data matrix. The relative abundance of Glomeromycota was less in the Dutch VM site than in OP. No obvious effect of the fungicide treatments or the potato varieties was detectable at this fungal phylum level (Figure 2).

### Diversity Indicators of the Bacterial and Fungal Communities

The Simpson diversity of 16S rRNA gene SVs was generally very similar between varieties, sites, and years (Table 1). The cisgenic modification [DC vs. SM and Desirée isogen (DI)] had no effect. In contrast, at OP in 2014, the application of fungicides significantly increased bacterial diversity. However, this effect appeared to be sporadic as it neither occurred in the previous year nor at the VM field site.

**TABLE 1:** Simpson diversity indices of three marker gene PCR amplicon DNA sequences at experimental sites in Oak Park (OP), Ireland, and Valthermond (VM), Netherlands, from field plots with three different potato cultivars: Sapo Mira (SM), Desirée isogen (DI), and Desirée cisgen (DC).

Site	Year	Cultivar	Treatment	16S rRNA gene	*nirK* gene	Fungal ITS region
OP	2013	SM	Fungicides	0.99 ± 0.006^a^	0.98 ± 0.004^ac^	0.78 ± 0.152^a^
			No fungicides	0.99 ± 0.005^a^	0.98 ± 0.013^abcd^	0.82 ± 0.125^a^
		DI	Fungicides	0.99 ± 0.005^a^	0.98 ± 0.01^abc^	0.84 ± 0.196^a^
			No fungicides	0.99 ± 0.007^a^	0.98 ± 0.009^abc^	0.88 ± 0.102^a^
		DC	Fungicides	0.99 ± 0.003^ab^	0.98 ± 0.005^a^	0.89 ± 0.095^a^
			No fungicides	0.98 ± 0.008^a^	0.97 ± 0.01^bd^	0.88 ± 0.071^a^
	2014	SM	Fungicides	0.97 ± 0.005^c^	0.96 ± 0.022^deh^	0.89 ± 0.053^a^
			No fungicides	0.95 ± 0.024^cd^	0.95 ± 0.008^eh^	0.92 ± 0.015^a^
		DI	Fungicides	0.97 ± 0.007^c^	0.96 ± 0.016^de^	0.91 ± 0.03^a^
			No fungicides	0.96 ± 0.017^cd^	0.95 ± 0.053^de^	0.89 ± 0.098^a^
		DC	Fungicides	0.97 ± 0.005^c^	0.92 ± 0.067^efgh^	0.88 ± 0.055^a^
			No fungicides	0.94 ± 0.035^d^	0.89 ± 0.041^fg^	0.87 ± 0.05^a^
VM		SM	Fungicides	0.99 ± 0.004^a^	0.82 ± 0.028^g^	0.92 ± 0.019^a^
			No fungicides	0.99 ± 0.005^a^	0.96 ± 0.022^deh^	0.92 ± 0.039^a^
		DI	Fungicides	0.99 ± 0.001^b^	0.86 ± 0.05^fg^	Not applicable
			No fungicides	0.99 ± 0.003^a^	0.91 ± 0.061^bcdefh^	Not applicable
		DC	Fungicides	0.99 ± 0.004^a^	0.88 ± 0.075^fgh^	0.92 ± 0.025^a^
			No fungicides	0.99 ± 0.003^a^	0.96 ± 0.036^abcdeh^	0.94 ± 0.011^a^

*Experimental plots either received fungicides or not, as indicated. Groups within a column not sharing the same letter are significantly different (paired Wilcoxon test and Benjamini–Hochberg adjustment, *p* < 0.05). ITS, internal transcribed spacer.*

For the *nirK* gene, no significant differences were detected for the cisgenic modification. The fungicides increased the diversity at OP in 2013 for DC, but this was not seen in 2014 at the OP or VM sites. At the VM site, and only there, a significantly lower diversity was detected with the SM variety, but the trend of lower diversity in response to fungicides was also seen for both Desirée varieties (Table 1).

Simpson diversity of the fungal ITS sequences was neither affected by the cisgenic modification, the variety, the site of cultivation, nor the application of the fungicides.

### Impact of Site, Year, Variety, Fungicides, and Cisgenic Modification on the Microbial Community Compositions

**FIGURE 3:**
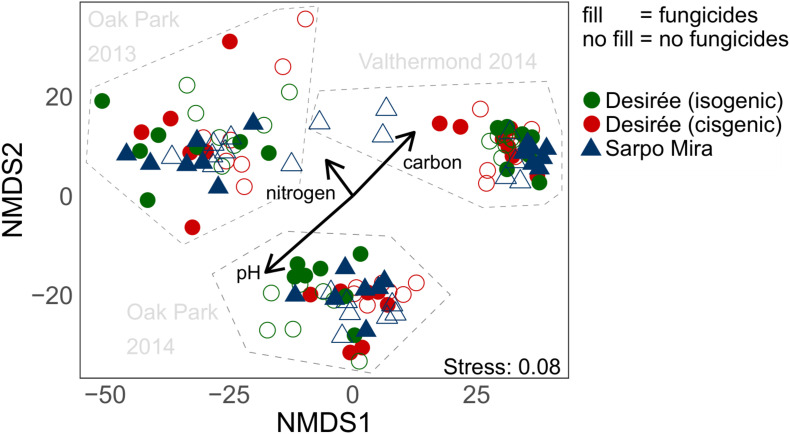
Non-metric multidimensional scaling (NMDS) of 16S rRNA sequence variances (SVs) in all soil samples. SVs with relative abundances lower than 0.05% were removed, which left 359 SVs that accounted for 86% of total sequences. Vector fitting was used to depict the influence of the environmental variables soil pH, nitrogen, and organic C content on ordination results.

PERMANOVA and the NMDS plot from the 16S RNA SVs (Figure 3 and Table 2) showed that prokaryotic communities in the rhizosphere were separated by year and location. The separation of the OP and VM sites correlated with the amount of total soil organic C and pH values in opposite directions, while the variation in soil nitrogen had an impact on the annual difference seen at the OP site.

**TABLE 2:** Results from PERMANOVA and variance partitioning, Oak Park (OP), Ireland, and Valthermond (VM), Netherlands.

Gene/Region	Site	Year	Factor	PERMANOVA		Variance partitioning	
				R-squared	*p*-value	% explanation	*p*-value
16S rRNA	All	All	Year	**0.2744**	0.001	**19.7**	0.001
			Location	**0.3001**	0.001	**22.2**	0.001
	OP	2013	Cultivar	**0.0875**	0.006	**4.4**	0.002
			Treatment	**0.0618**	0.002	**4.1**	0.001
		2014	Cultivar	**0.1331**	0.001	**9.1**	0.001
			Treatment	0.0290	0.18	0.7	0.079
	VM	2014	Cultivar	**0.0823**	0.001	**3.7**	0.002
			Treatment	0.0341	0.067	1.1	0.057
*nirK*	All	All	Year	**0.1276**	0.001	**8.2**	0.001
			Location	**0.2720**	0.001	**22.8**	0.001
	OP	2013	Cultivar	**0.0929**	0.005	**4.8**	0.003
			Treatment	0.0376	0.081	1.5	0.064
		2014	Cultivar	**0.0741**	0.024	**2.7**	0.02
			Treatment	0.0226	0.507	0	0.473
	VM	2014	Cultivar	0.0847	0.101	**4.8**	0.025
			Treatment	**0.1599**	0.001	**15.2**	0.001
Fungal ITS region	All	2014	Year	na	na	na	na
			Location	**0.3107**	0.001	na	na
	OP	2013	Cultivar	**0.0978**	0.001	**5.3**	0.002
			Treatment	0.0217	0.598	0	0.529
		2014	Cultivar	0.0575	0.126	1	0.131
			Treatment	0.0270	0.263	0.3	0.270
	VM	2014	Cultivar	0.0657	0.069	2.1	0.056
			Treatment	0.0523	0.267	0.7	0.257

*For the internal transcribed spacer (ITS) region, comparisons were only made within each year and between locations in 2014. Significant values are highlighted in bold. na, not applicable.*

Variance partitioning revealed that the variables year and location explained 20% and 22% of the variation in the bacterial and fungal community composition data (Table 2). PERMANOVA also indicated for both sites and years of sampling significant differences of 16S rRNA gene compositions between varieties. But these differences were minor, as they only explained 4–9% of the variance. Only in OP in 2013, but not in OP or VM in 2014, the application of fungicides had a significant effect, which, for that particular case, explained 4% of the community variation.

**FIGURE 4:**
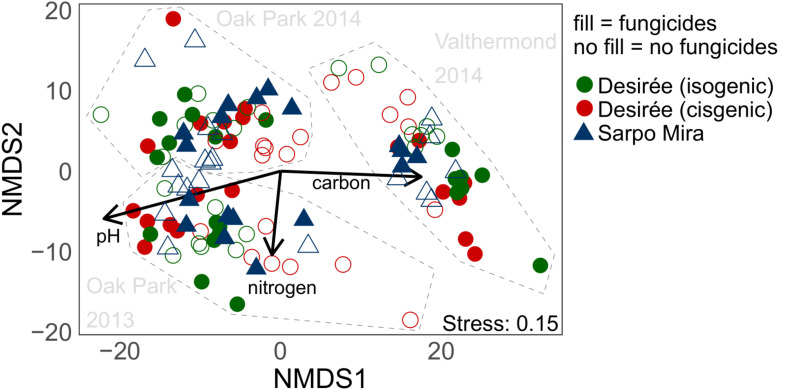
Non-metric multidimensional scaling (NMDS) of *nirK* gene fragment sequence variances (SVs) in all soil samples. SVs with relative abundances lower than 0.1% were removed, which left 174 SVs that accounted for 75% of total sequences. The environmental variables pH, soil nitrogen, and organic C content were fitted onto to the ordination by using vector fitting.

In accordance with the 16S rRNA gene results, bacterial communities differentiated by their *nirK* genes also formed distinct clusters according to the site and year of sampling the potato rhizospheres in the NMDS plot (Figure 4). The differences between the communities from OP in the two different years, however, were not equally strongly pronounced as for the 16S rRNA genes (Figure 4). Again, pH and soil C separated the sites from each other, while N gradients followed the annual variation of the OP soils.

The significant impact of site and year on the composition of the *nirK*-defined bacterial communities was also confirmed by PERMANOVA, and variance partitioning indicated that the site was more important than the year, explaining 23% of the variation, while annual variation explained 8% (Table 2). A significant effect of the variety was seen in OP for both years but not in VM. In contrast, the fungicide applications affected the *nirK* communities in VM but not in OP rhizosphere soils. The effect was comparably strong, as it explained 15% of the variation in the data.

**FIGURE 5:**
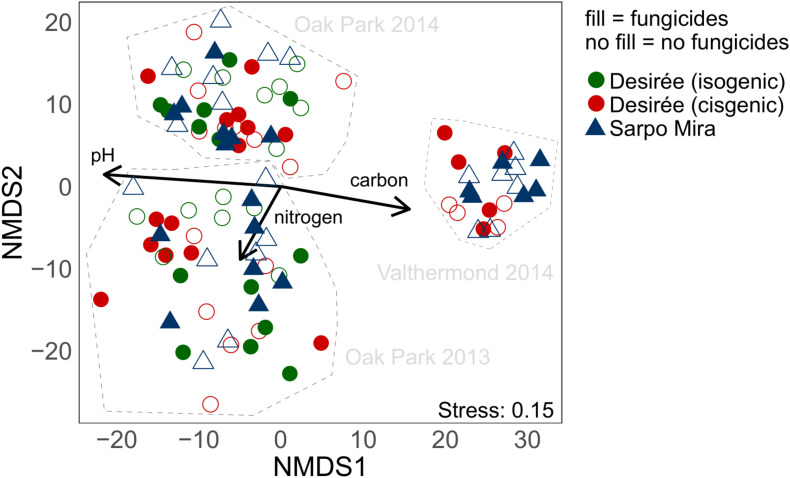
Non-metric multidimensional scaling (NMDS) of internal transcribed spacer (ITS) region sequence variances (SVs) in all soil samples. SVs with relative abundances lower than 0.1% were removed, which left 114 SVs that accounted for 89% of total sequences. Vectors are shown to depict the influence of the environmental variables pH, soil nitrogen, and organic C content. Each year was analyzed separately to exclude run variation biases from the two separate sequencing runs.

Regarding differences in fungal community structure, NMDS revealed a clear separation between samples from Ireland and the Netherlands in 2014 (Figure 5). This separation was confirmed by PERMANOVA. As for *nirK* and 16S rRNA gene-based analyses, pH and soil organic C correlated in opposite directions, driving the separation between sites. An effect of variety was only found in Irish samples in 2013, which explained 5% of the variation in the data (Table 2). The application of the fungicides had no detectable impact on the fungal community composition in the rhizosphere neither by NMDS nor by PERMANOVA analyses.

### Indication of Responsive Taxa (Sequence Variants)

A differential abundance analysis was performed to identify SVs among the datasets targeting the 16S rRNA gene (Bacteria, Archaea), the *nirK* gene, and the fungal ITS sequences. Independent of the year and site of cultivation, no significant differences were found between samples of the cisgenic DC and isogenic DI varieties. However, when both Desirée versions, DI and DC, were combined and then compared to SM, a total of 12 responsive 16S rRNA gene-based SVs from four different phyla were identified, but only at the OP site in 2013 (Table 3). Seven SVs were Proteobacteria, and six of them were Alphaproteobacteria; together, they represented 1.09% of the total relative abundance in the rhizosphere of Desirée (DI and DC), while they only represented 0.15% with SM, indicating variety differences. The higher relative abundance of Proteobacteria with DI and DC compared to SM was also seen with *nirK* genes: Four differentially abundant SVs, three of them *Phyllobacteriaceae*, represented 8.7% of all *nirK* sequences in DC and DI (combined), while they only represented 1.4% in the rhizosphere of SM. Similarly, a total of four fungal ITS SVs of significantly different abundance between DI and DC compared to SM were detected at the OP site in 2013, three ascomycetes and one basidiomycete. In all cases, the SVs were more abundant with Desirée (DI, DC), with a total relative abundance of 8.4%, while SM only represented 1.1%. In contrast, the differentially abundant SVs assigned to Actinobacteria showed together no consistent increase with Desirée compared to SM.

**TABLE 3:** List of sequence variances (SVs) from potato rhizospheres and their corresponding taxonomic affiliation that were differentially abundant between cultivars Sarpo Mira (SM) and Desirée (isogenic, DI, and cisgenic, DC, combined).

Gene target	Phylum or Class	Closest phylogenetic relative	SV number	Effect size	*P*-value	Average relative abundance % ± SD	
						DI and DC	SM
16S rRNA gene	Alphaproteobacteria	*Micropepsaceae*	SV343	−0.93	0.018	0.12 ± 0.13	0.004 ± 0.01
		*Rhizobiaceae*	SV98	−1.08	0.014	0.16 ± 0.15	0.02 ± 0.08
		*Xanthobacteraceae*	SV93	−1.18	0.009	0.25 ± 0.2	0.03 ± 0.1
		*Xanthobacteraceae*	SV153	−1.13	0.014	0.19 ± 0.15	0.04 ± 0.11
		*Rhodobacteraceae*	SV650	−0.83	0.041	0.09 ± 0.12	n.d.
		*Sphingomonadaceae*	SV203	−1.01	0.033	0.17 ± 0.13	0.06 ± 0.15
	Gammaproteobacteria	*Moraxellaceae*	SV570	−1.22	0.008	0.11 ± 0.1	nd
	Actinobacteria	Gaiellales	SV84	0.79	0.040	0.42 ± 0.15	0.51 ± 0.13
		*Gaiellaceae*	SV58	0.94	0.008	0.57 ± 0.21	0.75 ± 0.2
		*Microbacteriaceae*	SV71	0.82	0.024	0.44 ± 0.13	0.56 ± 0.18
		*Streptomycetaceae*	SV7	−0.91	0.036	1.96 ± 1.31	0.25 ± 0.4
	Bacteroidetes	*Chitinophagaceae*	SV275	−1.21	0.013	0.24 ± 0.25	0.04 ± 0.14
Fungal ITS region	Ascomycota	*Chaetomiaceae*	SV11	−1.46	0.001	4.67 ± 3.26	0.6 ± 1.74
		*Microascales*	SV30	−1.36	0.001	1.54 ± 1.26	0.23 ± 0.69
		*Togniniaceae*	SV62	−0.88	0.021	0.55 ± 0.53	0.09 ± 0.34
	Basidiomycota	*Piskurozymaceae*	SV16	−1.01	0.005	1.36 ± 1.19	0.14 ± 0.35
*nirK* gene	Alphaproteobacteria	*Phyllobacteriaceae*	SV10	−1.58	0.003	5.65 ± 4.13	0.74 ± 2.57
		*Phyllobacteriaceae*	SV106	−1.67	0.003	0.98 ± 0.78	0.08 ± 0.31
		*Phyllobacteriaceae*	SV226	−0.83	0.044	0.45 ± 0.42	0.04 ± 0.14
	Gammaproteobacteria	uncultured (1062)	SV45	−1.25	0.008	1.63 ± 1.39	0.57 ± 2.14

*SVs were based either on DNA sequences of prokaryotic 16S rRNA genes and of fungal internal transcribed spacer (ITS) regions or on deduced amino acid sequences of *nirK* genes, respectively. Data obtained from samples taken in Oak Park (OP) in 2013. The significance test was based on Welch’s *t-*test with Benjamini–Hochberg correction.nd, not detected.*

Differentially abundant taxa (SVs) were also detected when comparing the conventional treatment of using fungicides (see section “Materials and Methods”) to controls without fungicides. The dataset included in these analyses comprised all SVs identified from all three potato varieties (DI, DC, SM) and were analyzed separately for OP 2013, OP 2014, and VM 2014. At OP in 2013, A total of six different 16S rRNA gene-defined SVs were detected (Table 4). In the presence of fungicides, all bacteria, except for one representative of *Solirubrobacterales* (Actinobacteria), were less abundant: together, they represented 1.7%, while in controls without fungicides, they represented 2.3%. In addition, three *nirK* SVs were identified in VM in 2014. Here, a much higher relative prevalence in the presence of fungicides was detected for two *nirK*-defined SVs, both Phyllobacteriaceae, which together represented 44.8% of all *nirK* sequences, while they only represented 23.2% in controls without fungicides. No fungicide-responsive fungal SV was identified at any of the sites and years (Table 4).

**TABLE 4:** Sequence variances (SVs) and their corresponding taxonomic affiliations that were differentially abundant in rhizospheres of potatoes treated with fungicides or non-treated controls.

Site	Gene target	Phylum or Class	Family	SV number	Effect size	*P*-value	Average relative abundance % ± SD	
							With fungicides	No fungicides
OP	16S rRNA gene	Alpha-proteobacteria	*Sphingomonadaceae*	SV106	0.85	0.0009	0.40 ± 0.10	0.45 ± 0.19
			*Caulobacteraceae*	SV121	0.97	0.0009	0.33 ± 0.15	0.37 ± 0.13
			*Devosiaceae*	SV32	1.25	0.0001	0.44 ± 0.20	0.69 ± 0.32
			*Xanthobacteraceae*	SV49	1.21	0.0002	0.51 ± 0.21	0.70 ± 0.26
		Actinobacteria	*Nocardiaceae*	SV430	5.73	0.0014	0.02 ± 0.05	0.10 ± 0.07
			*Solirubrobacterales*	SV483	5.77	0.0008	0.13 ± 0.08	0.03 ± 0.07
VM	*nirK* gene	Alpha-proteobacteria	*Phyllobacteriaceae*	SV1	−0.74	0.0361	22.97 ± 7.00	13.4 ± 9.30
			*Phyllobacteriaceae*	SV3	−0.77	0.0361	21.85 ± 10.34	9.77 ± 8.67
			*Phyllobacteriaceae*	SV88	−0.86	0.0356	1.27 ± 1.10	0.26 ± 0.40

*SVs were defined by DNA sequences from prokaryotic 16S rRNA genes, fungal internal transcribed spacer (ITS) regions, and deduced amino acid sequences of bacterial *nirK* genes amplified from DNA collected in Oak Park (OP) in 2013 and Valthermond (VM) in 2014. The significance test was based on Welch’s *t-*test with Benjamini–Hochberg correction.*

## Discussion

Due to the release of root exudates and root cells, the rhizosphere provides energy-rich carbon sources to soil microorganisms, resulting in microbial growth and consequently increased biomass compared to other soil compartments (Dennis et al., 2010; Haichar et al., 2014). The composition and diversity of the microbial community assembly in the rhizosphere are affected by both, the properties of the plant-released products and of the soils in which the plants grow, the latter representing a major source from which particular members of the microbial community are enriched (Berg and Smalla, 2009; Wang et al., 2018). The composition of the indigenous soil microbial community again is a result of the physicochemical properties that have evolved at different time scales, on one side over a long period during pedogenesis and, on the other, at a shorter term in response to local weather conditions and agricultural management, the latter including crop rotations or the use of pesticides (Li et al., 2012; Yang et al., 2012; Babin et al., 2019; Shi et al., 2019). The rhizosphere itself can modify the physicochemical soil properties, e.g., by altering the pH through exudation of organic acids (Henry et al., 2008). In this study, however, the physicochemical properties of the rhizosphere soils were neither specifically affected by the cisgenic modification nor by the variety or the application of fungicides.

The contrasting soil properties at the sites in Ireland (OP) and the Netherlands (VM), which were tangible by differences in pH, organic C, and the C/N ratio, resulted in clearly distinguishable rhizosphere-inhabiting microbial communities, thus, demonstrating that the analyses as applied here were sensitive enough for detecting alterations of microbial abundance and diversity. Acidobacteria were more abundant at the site in VM than in OP, which can be explained by their preference for a lower soil pH (Jones et al., 2009; Griffiths et al., 2011). Furthermore, the fungal abundance was higher in VM, which could as well be an outcome of the lower pH or, possibly also, a surplus of C in VM compared to OP (Venneman et al., 2019). The two field sites also strongly affected the diversity of bacteria providing *nirK* genes to the microbial community: *Sinorhizobium* sp. dominated in OP while *M. ciceri* dominated in VM, indicating that the same functions were provided by different taxa. Factors, i.e., pH, soil organic C, or water saturation, were all identified as being of paramount importance for shaping soil microbial communities in larger biogeographical studies (Fierer and Jackson, 2006; Griffiths et al., 2011; Hamonts et al., 2013; Azziz et al., 2017; Szoboszlay et al., 2017).

Despite indistinguishable physicochemical soil properties between rhizospheres from the same site, some significant differences between the microbial communities could be picked up in this study, suggesting that they were caused by the potato roots or alterations due to fungicide treatments. At the Dutch site (VM), bacterial abundance was lower with fungicides, but this effect was not seen in OP, neither in 2013 nor in 2014. In fact, in 2014, the fungicide applications even had a slightly positive effect and bacterial abundance and also on bacterial diversity. However, the differences between microbial abundance linked to fungicides were minor and not consistent. They may have been a result of stochastic effects and should not be overweighted due to the lack of annual replication at the VM site. In an accompanying study on GM maize rhizosphere microbial communities, it was already demonstrated that individual differences between varieties and between a GM and non-GM maize lose significance when annually replicated across sites (Szoboszlay et al., 2019).

It may appear surprising that the fungicides applied in this study affected bacterial but not fungal parameters. The fungicides applied here were mainly targeting the pathogen *P. infestans*, which is a member of the Oomycetes and, thus, phylogenetically more closely related to algae Kingdom Chromista than to other fungal phyla subsumed in the Kingdom Fungi, the latter predominating in agricultural soil and crop rhizospheres (Mardanova et al., 2019; Hou et al., 2020). DNA sequencing in this study confirmed that the fungal community consisted almost exclusively of Ascomycota, Basidiomycota, and Glomeromycota (Hibbett et al., 2007). In addition to these three dominant fungal phyla, the universal primer used in this study also picked up individual sequences from a protist phylum. In contrast, Oomycetes were not detected, even though the ITS1 and ITS2 primers would have the specificity to also amplify Phytophthora and their relatives (Cooke and Duncan, 1997). Interestingly, in a parallel study, symptoms of *P. infestans* were detected at the respective sites in the same field experiment (Kessel et al., 2018), thus suggesting that the pathogen was highly enriched in the host plants compared to the soil compartment. Thus, the infection most likely originated from airborne fungal spores and not directly from the surrounding soil. While the fungicides strongly limited the disease incidence and thereby controlled the Oomycete pathogen (Kessel et al., 2018), it had no tangible off-target effects on fungi colonizing the potato rhizospheres, thus confirming that common fungi and Oomycetes may strongly vary in their susceptibility toward fungicides (Latijnhouwers et al., 2003).

In addition to the structural markers for bacterial and fungal diversity, this study included a functional marker by targeting the prokaryotic nitrite reductase. Due to root exudation of carbonaceous compounds and the nitrogen requirements of the growing crops, nitrogen is usually limited in crop rhizospheres. Thus, it was suspected that the spatial shortage of nitrogen created selective pressure on microbial communities involved in the soil nitrogen cycle (Kastl et al., 2015), and therefore, the nitrite reductase and its two alternative genetic versions *nirS* and *nirK* were chosen as markers. A quantitative dominance of *nirK* compared to *nirS* genes of approximately two orders of magnitude was found in potato rhizosphere soil at both sites OP and VM. Previously, a similar dominance of *nirK* vs. *nirS* was found with the same primers sets on five maize field sites across Europe (Szoboszlay et al., 2019), while, in contrast, sites across Turkey showed a dominance of *nirS* (Avsar and Aras, 2020). A preference for *nirS* at higher and *nirK* at lower pH values, as suggested in another study (Bowen et al., 2020), could not be confirmed.

Despite numerous studies on *nirS* and *nirK* abundance, their niche preference or indicator potential of their ratio in soil and rhizosphere remains poorly understood (Babic et al., 2008; Orlando et al., 2012; Azziz et al., 2017; Brin et al., 2019; Perez-Brandan et al., 2019). However, some studies already demonstrated that root exudates of crops affected gene abundance and expression of *nirK*- and *nirS*-bearing bacterial communities (Sharma et al., 2005; Hou et al., 2018; Achouak et al., 2019). Apparently, the providers of nitrite reductases can live in close contact with plant-released products. This supports our approach for selecting *nir* gene abundance and *nirK* genetic diversity as indicators. In the rhizosphere of Desirée, irrespective of cisgenic or not, in 2013, the abundance of *nirS* was stimulated in the presence of fungicide, but this effect was not confirmed by annual replication. Similarly, for *nirK* and only for the VM site (which lacked annual replication), fungicides stimulated their abundance, indicating enhanced denitrification potential. This was seen more pronounced for Desirée and significant only for the cisgenic version. Thus, even though this was a case of detecting a significant difference of the cisgenic modification, it is apparent that this reflected variation rather than a biological difference. For other chemical pesticides, effects on *nirS* and *nirK* abundance in the rhizosphere were also detected, but those were interpreted as being adverse due to declining copy numbers (Singh et al., 2015).

While all three genetic markers selected for this study for assessing the microbial diversity, i.e., the prokaryotic 16S rRNA and *nirK* genes and the fungal ITS sequences showed some individual responses to field site, variety, or year of cultivation, the multivariate statistical analyses revealed that pH and soil organic C were the best explanatory variables for the distinct communities and that soil nitrogen was also influential. Fungal communities were distinct between sites but unaffected by any of the treatments, while the prokaryotic markers showed some responses to treatments, which however, were either not consistent across the two potato varieties or not confirmed by annual replication. With the major focus of this study on the impact of cisgenic modification, none of the markers indicated consistent effects. Considering that majority of transgenic GM plants studied to date have given little indication of unintended effects on soil microbial communities in comparative studies (Hannula et al., 2014; Nicolia et al., 2014; Kolseth et al., 2015; Young et al., 2017), the cisgenic modification analyzed here suggests, based on the case of the late blight-resistant Desirée, that there is no specific concern associated with this cisgenic modification.

## Conclusion

This study demonstrates with a highly sensitive molecular detection technique that soil bacterial and fungal communities, both likely to be influenced by plant-released products in the rhizosphere, respond to field site conditions, to annual variation, and, sporadically, to fungicides and potato varieties, but not to a cisgenic modification of a widely cultivated potato variety. Parallel studies at the same field sites using the same experimental design failed to detect tangible effects of this cisgenic potato on the soil nematode community structure (Ortiz et al., 2016) and on arthropod diversity (Lazebnik et al., 2017). Together, these studies demonstrate a lack of effects on non-target organisms and indicate no environmental risks associated with cultivating this cisgenic potato.

## Data Availability Statement

The datasets presented in this study can be found in online repositories. The names of the repository/repositories and accession number(s) can be found below: https://www.ebi.ac.uk/ena, PRJEB39718.

## Author Contributions

CT initiated this work, supervised it, co-analyzed all data, and wrote the manuscript. AN organized the field sampling campaigns, collected the soil samples, performed all wet-lab work and a first analysis of the data, and participated in preparing this manuscript. SK selected and applied the bioinformatic pipeline and the statistical data evaluations and wrote the manuscript. EM supervised the fungal sequence analyses and the field study in Oak Park with VC coordinating sample collection and field management. LL and GK set up the field experiments and supervised the field study in Valthermond. All authors contributed to the article and approved the submitted version.

## Conflict of Interest

The authors declare that the research was conducted in the absence of any commercial or financial relationships that could be construed as a potential conflict of interest.

## Supplementary Material

Supplementary Table 1 Primer sequences and probes used in qPCR and Illumina sequencing.

Supplementary Table 2 Properties of rhizosphere soils collected for microbial community analyses from potatoes grown at the experimental sites in Oak Park (OP) and Valthermond (VM). Cultivars included Desirée isogenic (DI), Desirée cisgenic (DC), and Sarpo Mira (SM). The experimental plots were either treated with fungicides or not. Each treatment and cultivar was replicated at each site seven times.
