# Supplementary material for: No Tangible Effects of Field-Grown Cisgenic Potatoes on Soil Microbial Communities
Source: Front Bioeng Biotechnol. 2020 Nov 3;8:603145. doi: 10.3389/fbioe.2020.603145 (PMC7670967; doi:10.3389/fbioe.2020.603145)
Supplement: Supplementary Table 1 — Primer sequences and probes used in qPCR and Illumina sequencing. [file Table_1.docx]

**Table S1:** Primer sequences and probes used in qPCR and Illumina sequencing

| **Target** | **Length** | **Primer/Probe** | **Reference** |
| --- | --- | --- | --- |
| qPCR | | | |
| Bacterial 16S rRNA | 468 bp | BAC338F (5’-ACTCCTACGGGAGGCAG-3’)  BAC516F (5’-TGCCAGCAGCCGCGGTAATAC-3’)  BAC805R (5’-GACTACCAGGGTATCTAATCC-3’) | Yu et al., 2005 |
| Archaeal 16S rRNA | 273 bp | ARC787F (5’- ATTAGATACCCSBGTAGTCC-3’)  ARC915F (5’- AGGAATTGGCGGGGGAGCAC-3’)  ARC1059R (5’-GCCATGCACCWCCTCT-3’) |  |
| Fungal ITS | 300-500 bp | NSI1 (5’- GATTGAATGGCTTAGTGAGG-3’)  58A2R (5’- CTGCGTTCTTCATCGAT-3’) | Martin, 2005 |
| *nirS* | 426 bp | nirSCd3a-F (5’- AACGYSAAGGARACSGG-3’)  nirSR3cd-R (5’- GASTTCGGRTGSGTCTTSAYGAA -3’) | Kandeler et al., 2006 |
| *nirK* | 165 bp | nirK876-F (5’- ATYGGCGGMVAYGGCGA -3’)  nirK1040-R (5’- GCCTCGATCAGRTTRTGGTT -3’) | Henry et al., 2004 |
| Gene-specific primers used for Illumina sequencing | | | |
| Bacteria | - | S-D-Bact-0785-a-A-21 (GACTACHVGGGTATCTAATCC) | Klindworth et al., 2013 |
| Archaea | - | S-D-Arch-0519-a-S-15 (CAGCMGCCGCGGTAA) |  |
| *nirS* | - | F1aCu (ATCATGGTSCTGCCGCG) | Throbäck et al, 2004 |
| *nirK* | - | *nirK*1040R (GCCTCGATCAGRTTRTGGTT) |  |
| Fungi | - | ITS1F (CTTGGTCATTTAGAGGAAGTAA)  ITS2 (GCTGCGTTCTTCATCGATGC) | Gardes and Burns, 1993; Bousquet et al., 2006 |

**References**

Bousquet P, Ciais P, Miller JB, et al. (2006) Contribution of anthropogenic and natural sources to atmospheric methane variability. Nature 443: 439-443.

Gardes M & Bruns TD (1993) ITS primers with enhanced specificity for basidiomycetes - application to the identification of mycorrhizae and rusts. Molecular Ecology 2: 113-118.

Henry S, Baudoin E, López-Gutiérrez JC, Martin-Laurent F, Brauman A & Philippot L (2004) Quantification of denitrifying bacteria in soils by nirK gene targeted real-time PCR. Journal of microbiological methods 59: 327-335.

Kandeler E, Deiglmayr K, Tscherko D, Bru D & Philippot L (2006) Abundance of narG, nirS, nirK, and nosZ genes of denitrifying bacteria during primary successions of a glacier foreland. Applied and environmental microbiology 72: 5957-5962.

Klindworth A, Pruesse E, Schweer T, Peplies J, Quast C, Horn M & Glockner FO (2013) Evaluation of general 16S ribosomal RNA gene PCR primers for classical and next-generation sequencing-based diversity studies. Nucleic acids research 41: e1.

Martin KJ & Rygiewicz PT (2005) Fungal-specific PCR primers developed for analysis of the ITS region of environmental DNA extracts. BMC microbiology 5: 28.

Throbäck IN, Enwall K, Jarvis A & Hallin S (2004) Reassessing PCR primers targeting nirS, nirK and nosZ genes for community surveys of denitrifying bacteria with DGGE. Fems Microbiol Ecol 49: 401-417.

Yu Y, Lee C, Kim J & Hwang S (2005) Group-specific primer and probe sets to detect methanogenic communities using quantitative real-time polymerase chain reaction. Biotechnology and Bioengineering 89: 670-679.
