# Supplementary material for: No Tangible Effects of Field-Grown Cisgenic Potatoes on Soil Microbial Communities
Source: Front Bioeng Biotechnol. 2020 Nov 3;8:603145. doi: 10.3389/fbioe.2020.603145 (PMC7670967; doi:10.3389/fbioe.2020.603145)
Supplement: Supplementary Table 2 — Properties of rhizosphere soils collected for microbial community analyses from potatoes grown at the experimental sites in Oak Park (OP) and Valthermond (VM). Cultivars included Desirée isogenic (DI), Desirée cisgenic (DC), and Sarpo Mira (SM). The experimental plots were either treated with fungicides or not. Each treatment and cultivar was replicated at each site seven times. [file Table_2.docx]

**Table S2:** Properties of rhizosphere soils collected for microbial community analyses from potatoes grown at the experimental sites in Oak Park (OP) and Valthermond (VM). Cultivars included Desirée isogenic (DI), Desirée cisgenic (DC), and Sarpo Mira (SM). The experimental plots were either treated with fungicides or not. Each treatment and cultivar was replicated at each site seven times

| Site | Year | Cultivar | Treatment | pH | Organic C  mg g^-1^ soil | Total N  mg g^-1^ soil | CN-ratio |
| --- | --- | --- | --- | --- | --- | --- | --- |
| OP | 2013 | DI | fungicide | 7.0 ± 0.2 | 52.7 ± 6.6 | 4.33 ± 0.44 | 12.3 |
|  |  |  | no fungicide | 6.8 ± 0.4 | 46.4 ± 4.7 | 3.90 ± 0.36 | 11.9 |
|  |  | DC | fungicide | 6.9± 0.3 | 54.3 ± 17.0 | 4.48 ± 0.71 | 12.0 |
|  |  |  | no fungicide | 6.5 ± 0,5 | 49.7 ± 11.2 | 4.36 ± 0.65 | 11.3 |
|  |  | SM | fungicide | 7.0 ± 0.2 | 49.9 ± 9.6 | 4.20 ± 0.38 | 11.8 |
|  |  |  | no fungicide | 7.0 ± 0.2 | 40.1 ± 6.4 | 3.80 ± 0.44 | 10.5 |
|  | 2014 | DI | fungicide | 6.9 ± 0.7 | 48.2 ± 10.7 | 3.47 ± 0.51 | 12.9 |
|  |  |  | no fungicide | 7.0 ± 0.2 | 43.4 ± 11.7 | 3.55 ± 0.65 | 12.2 |
|  |  | DC | fungicide | 6.8 ± 0.4 | 41.0 ± 4.8 | 3.45 ± 0.28 | 11.9 |
|  |  |  | no fungicide | 6.7 ± 0.6 | 37.7 ± 6.7 | 3.24 ± 0.47 | 12.3 |
|  |  | SM | fungicide | 6.7 ± 0.7 | 36.9 ± 9.3 | 3.06 ± 0.52 | 12.1 |
|  |  |  | no fungicide | 7.1 ± 0.6 | 53.2 ± 8.2 | 3.89 ± 0.48 | 13.7 |
| VM | 2014 | DI | fungicide | 4.6 ± 0.2 | 89.6 ± 2.3 | 3.73 ± 0.94 | 24.0 |
|  |  |  | no fungicide | 4.7 ± 0.1 | 88.5 ± 1.9 | 3.61 ± 0.87 | 24.7 |
|  |  | DC | fungicide | 4.6 ± 0.2 | 96.0 ± 3.1 | 4.01 ± 1.28 | 24.0 |
|  |  |  | no fungicide | 4.8 ± 0.2 | 86.7 ± 2.4 | 3.65 ± 0.93 | 23.7 |
|  |  | SM | fungicide | 4.5± 0.1 | 88.3 ± 1.8 | 3.72 ± 0.80 | 23.8 |
|  |  |  | no fungicide | 4.8 ± 0.1 | 73.3 ± 2.5 | 3.06 ± 1.06 | 24.0 |
